# Autologous Skin Cell Suspension Monotherapy Without Split-Thickness Skin Grafting for Deep Dermal Burns in Pediatric Patients: A Case Series

**DOI:** 10.7759/cureus.83210

**Published:** 2025-04-29

**Authors:** Junya Oshima, Yoshiaki Inoue, Kaoru Sasaki, Mitsuru Sekido

**Affiliations:** 1 Department of Plastic, Reconstructive, and Hand Surgery, University of Tsukuba, Tsukuba, JPN; 2 Department of Emergency and Critical Care Medicine, University of Tsukuba Hospital, Tsukuba, JPN; 3 Department of Plastic, Reconstructive, and Hand Surgery, University of Tsukuba, Ibaraki, JPN

**Keywords:** cell suspension, deep partial thickness burns, pediatric burns, recell, skin graft

## Abstract

RECELL® autologous cell harvesting device (AVITA Medical, Inc., Santa Clarita, California, United States) is a non-cultured autologous skin cell suspension (ASCS) device that rapidly processes a small sample of the patient’s own skin into a cell suspension that can be sprayed onto large burn wounds. The ASCS system is considered to be effective for treating burns in children, especially because it minimizes new scars caused by skin harvesting; however, there are very few reports on ASCS monotherapy without autologous split-thickness skin grafting in patients under three years of age. This report describes three cases of deep dermal burn in patients under the age of three years treated with ASCS monotherapy. The cases highlight the advantages of ASCS monotherapy, including expedited healing, reduced burden of wound care, and minimized scarring from skin harvesting. In ASCS monotherapy, challenges remain, including the fragility of the engrafted cells, the small but unavoidable need for skin harvesting, postoperative scarring, and high treatment costs. This report highlights the need for ongoing accumulation of case studies to refine treatment protocols and emphasizes the importance of extended follow-up to evaluate long-term outcomes, including the impact of growth on scar formation.

## Introduction

The treatment goals for pediatric burn wounds are acute wound epithelialization and minimization of long-term scarring. In pediatric patients, the use of autologous skin grafts for deep partial burns that undergo epithelialization with conservative treatment remains controversial [1]. RECELL^®^ autologous cell harvesting device (AVITA Medical, Inc., Santa Clarita, California, United States) is a non-cultured autologous skin cell suspension (ASCS) device that rapidly processes a small sample of the patient’s own skin into a cell suspension that can be sprayed onto large burn wounds [2]. The ASCS system, which received approval from Japan’s Pharmaceuticals and Medical Devices Agency in February 2022, is considered to be effective for treating burns in children, especially because it minimizes new scars caused by skin harvesting; however, there are very few reports on ASCS monotherapy without autologous skin grafting in patients under three years of age. Therefore, the purpose of the current case series was to investigate cases of pediatric burns treated at our hospital with ASCS monotherapy and without split-thickness skin grafting.

## Case presentation

This report describes the cases of three pediatric burn patients who were treated with ASCS alone, without skin grafting, at our hospital between January and August 2023. The medical records included the following information: age, gender, mechanism of burn, total burn area, superficial dermal burn (SDB) area, deep dermal burn (DDB) area, days between burn injury and ASCS application, number of sedated dressing changes, length of the intensive care unit (ICU) stay, length of hospital stay, time until wound healed, steroid tape start time, duration of use of steroid tape, duration of follow-up, and maximum Vancouver scar scale (VSS) at final visit. The Vancouver scar scale is an objective and verified approach for characterizing burn scars and includes a summary of the scar’s coloration (0-2), vascularity (0-3), pliability (0-5), and height (0-3) [3]. Each category receives a score of 0 for normal skin. Healing was defined as the number of days post-burn injury at which the wound was considered closed, and the patient no longer required dressing changes.

ASCS procedure and postoperative treatment

The commercially available ASCS system kit (AVITA Medical) was used in all three cases included in this report. In this system, a 0.2 mm-thick skin sample is harvested from the normal tissue area using an electric dermatome. According to the manufacturer’s instructions, the cell expansion ratio is 1:80; so, for example, if the recipient’s burn area is 80 cm^2^, a skin sample of 1 cm^2^ is taken. After enzymatically treating the skin sample according to the manufacturer’s instructions, the epidermal cells are separated from the dermal layer using a scalpel and expanded with lactate solution. The cell suspension is then aspirated with a 5 ml syringe and sprayed onto the debrided burn wound using a VERSAJET^®^ Hydrosurgery System (Smith & Nephew plc, Watford, United Kingdom) [4]. After spraying, the wound is covered with SI-Aid silicone gel mesh dressing (ALCARE Co., Ltd., Tokyo, Japan) and gauze. The gauze is removed for the first time on the seventh day after the ASCS treatment, and thereafter replaced every one to two days until epithelialization is achieved. After epithelialization, if the wound thickened or inflammation persisted, steroid tape is applied [5,6].

Clinical data and outcomes

Case 1

**Figure 1:**
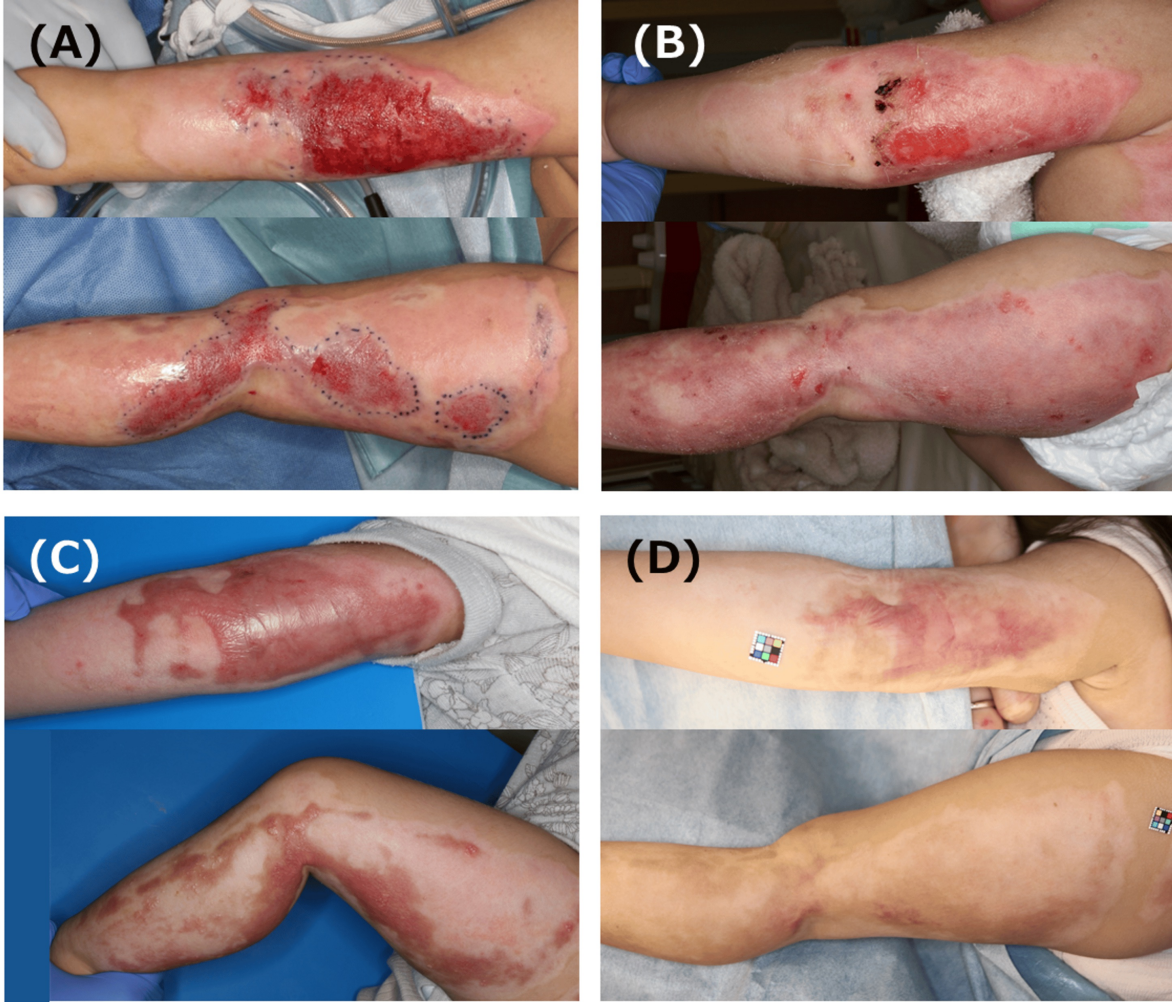
Clinical images of Case 1 A: Burn wound just before ASCS spray. The patient was splashed with boiling water and suffered burns to her left upper limb and left lower limb. ASCS was used 14 days post-injury for 5% DDB with prolonged spontaneous epithelialization. B: Findings on day 14 after ASCS treatment (28 days post injury). An epithelial defect remains in the olecranon. C: Findings 83 days post injury. The scar exhibited marked inflammation, and steroid tape was initiated. D: Findings 321 days post injury. The inflammation had generally disappeared, although some thickening remained in the elbow area. (A color chart for color correction is visible in the photo.） ASCS: autologous skin cell suspension

The patient, a one-year-old girl, had been splashed with boiling water and suffered burns to her left upper limb and left lower limb. ASCS was used 14 days after the injury for 5% DDB with prolonged spontaneous epithelialization (Figure 1A, 1B). Epithelialization of the olecranon was prolonged, and complete epithelialization was achieved at all sites 41 days after the injury. The wound had severe inflammation, and steroid tape application was started 83 days after the injury (Figure 1C). At the time of the final examination, the inflammation had generally disappeared, although some thickening remained in the elbow area (Figure 1D).

Case 2

**Figure 2:**
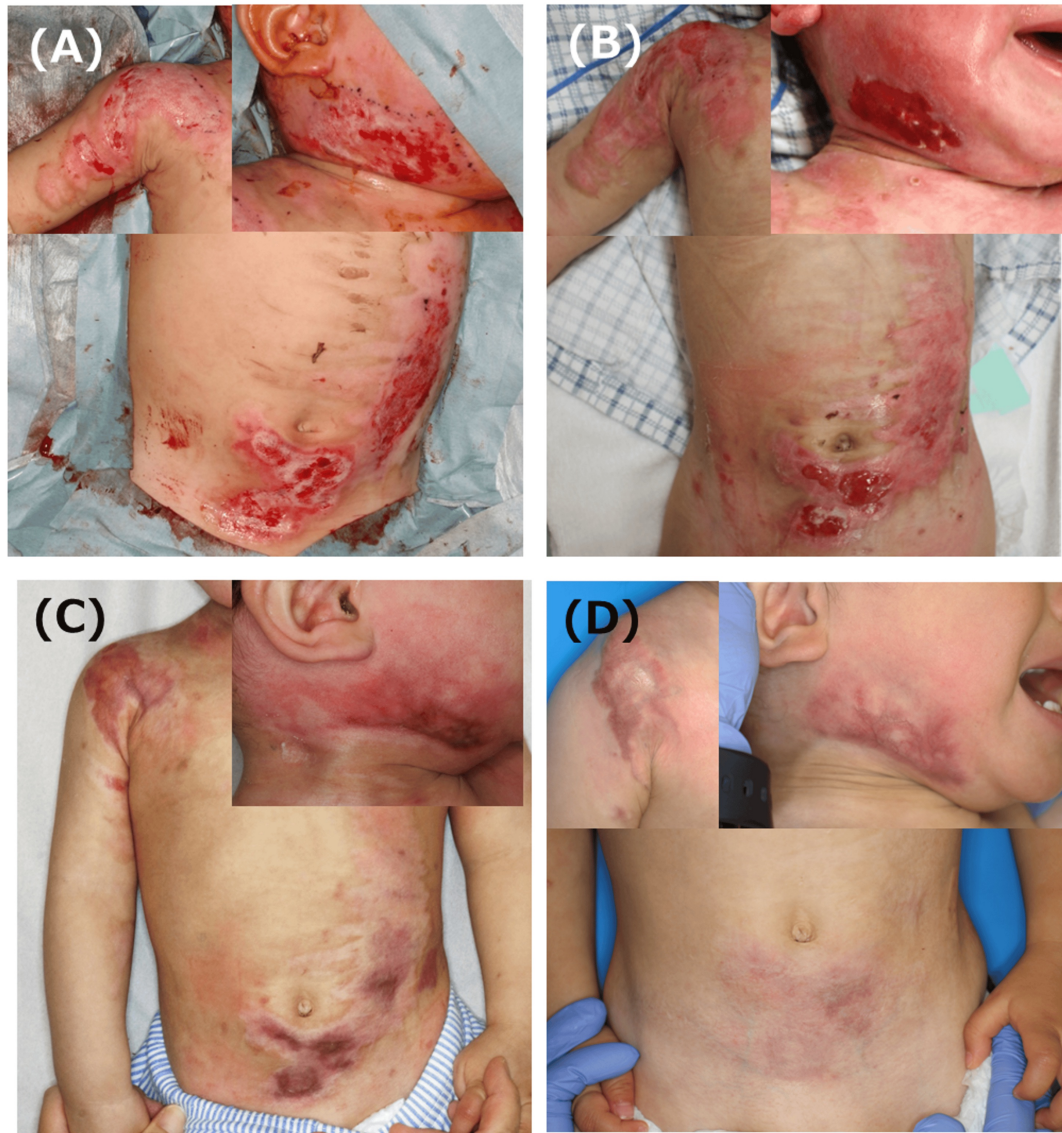
Clinical images of Case 2 A: Burn wound just before ASCS spray. The patient had been splashed with boiling water and suffered burns to his right lower jaw, right shoulder, and chest and abdomen. ASCS was used 10 days after injury for 5% DDB with prolonged spontaneous epithelialization. B: Findings on day 14 after ASCS treatment (24 days after injury). An epithelial defect remains in parts of the mandible and shoulder. C: Findings 54 days after injury. The scar had mild inflammation, and steroid tape application was initiated. D: Findings 257 days post injury. Redness remained on the lower jaw and shoulder, and follow-up is currently ongoing. ASCS: autologous skin cell suspension

The patient, an 11-month-old boy, had been splashed with boiling water and suffered burns to his right lower jaw, right shoulder, chest, and abdomen. ASCS was used 10 days after the injury for 5% DDB with prolonged spontaneous epithelialization (Figure 2A, 2B). Epithelialization in parts of the mandible and shoulder was prolonged, and complete epithelialization was achieved in all areas 44 days after the injury. Steroid tape application was started 54 days after the injury for areas with delayed healing (Figure 2C). At the time of the final examination, redness remained on the lower jaw and shoulder, and follow-up is currently ongoing (Figure 2D).

Case 3

**Figure 3:**
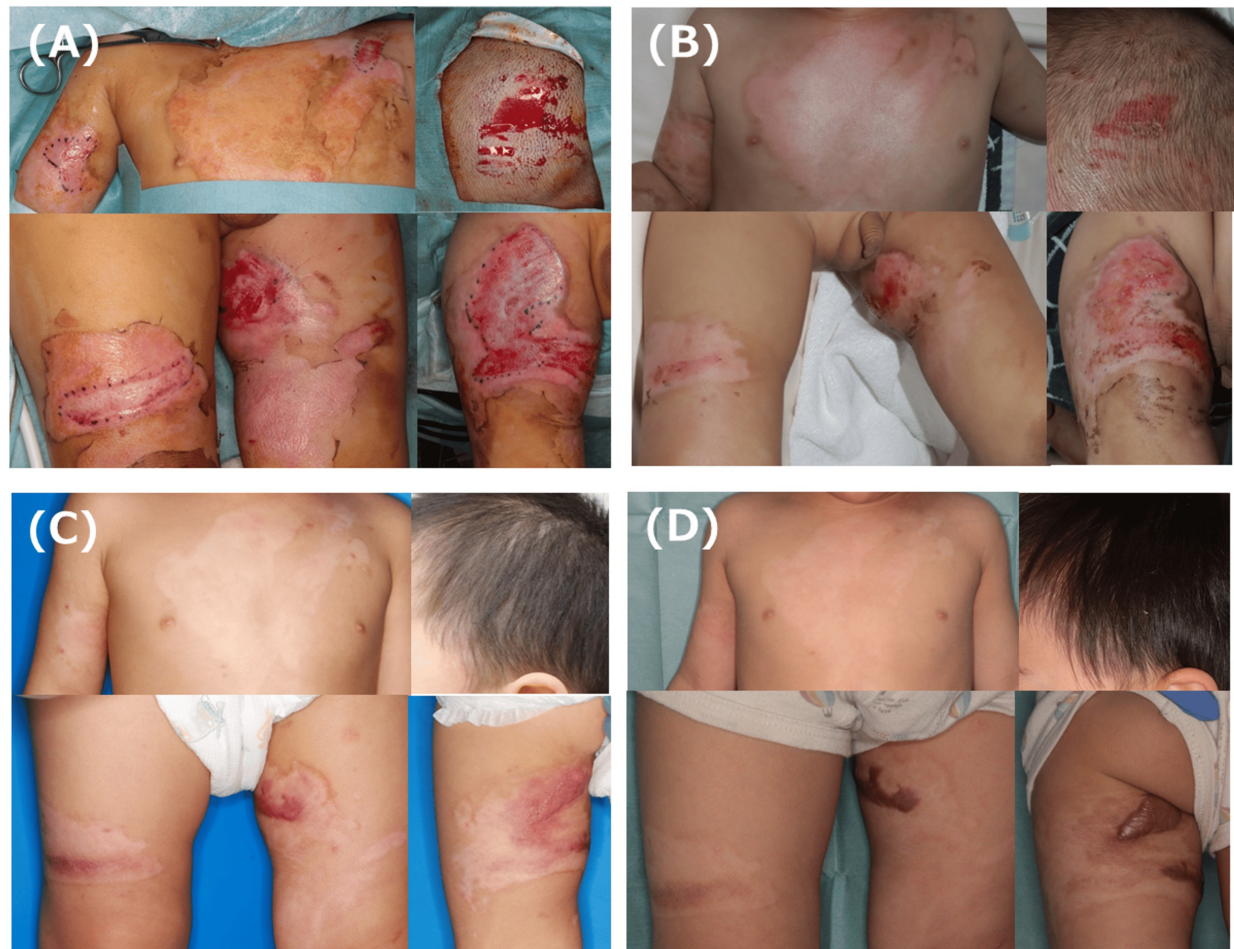
Clinical images of Case 3 A: Burn wound just before ASCS spray. The patient had been splashed with boiling water and suffered burns to the anterior chest, right upper arm, and both thighs. ASCS was used eight days after injury for 4% DDB with prolonged spontaneous epithelialization. B: Findings on day 14 after ASCS (22 days after injury). An epithelial defect remains in inner side of the left thigh C: Findings 94 days post injury. The scar had mild inflammation. Steroid tape application was not feasible because the patient would remove it himself. D: Findings 220 days post injury. Redness remained on the left thigh and follow-up is currently ongoing. ASCS: autologous skin cell suspension

The patient, a two-year-old boy, had been splashed with boiling water and suffered burns to the anterior chest, right upper arm, and both thighs. ASCS was used eight days after injury for 4% DDB with prolonged spontaneous epithelialization (Figure 3A, 3B). Epithelialization of the inner side of the left thigh was prolonged, and complete epithelialization was achieved in all areas 45 days after the injury. Steroid tape application was not feasible in this case because the patient would remove it himself (Figure 3C). At the final examination, there was redness on the inside of the left thigh, and follow-up is currently ongoing (Figure 3D).

A summary of the three cases is shown in Table 1. 

**Table 1:** Summary of the three cases of pediatric burns treated with ASCS monotherapy TBSA: total body surface area; ASCS: autologous skin cell suspension

Parameters	Case１	Case２	Case３
Age	1 year 2 months	11 months	2 years 8 months
Sex	Female	Male	Male
Mechanism of burn	Scald	Scald	Scald
TBSA (%)	15	15	22.5
Superficial dermal burn (%)	10	10	18.5
Deep dermal burn (%)	5	5	4
Interval between injury and ASCS application (days)	14	10	8
Number of sedated dressing changes	15	11	10
Length of stay in ICU (days)	17	11	14
Length of hospital stay (days)	31	26	22
Time until wound healed (days)	41	44	45
Interval between injury and steroid tape application (days)	83	54	N/A
Duration of use of steroid tape (months)	175	175	N/A
Length of follow-up (days)	321	257	220
Maximum Vancouver scar scale at healing	7	5	11

## Discussion

Treatment for DDB can be divided into two main approaches: skin grafting and conservative therapy. Skin grafting for DDB is common in adult treatment because it expedites the treatment period [7]; however, harvesting of a skin graft leads to further scarring. Furthermore, early skin grafting when the depth of the burn is uncertain may result in the harvesting of unnecessary grafts. Even if the skin is grafted early, the wound may thicken and contract due to growth, and there is a possibility that further surgery may be required [8]. In contrast, conservative treatment is typically considered for DDB in children, especially infants, who have naturally strong skin-healing capacity [1,7]. Opting for conservative treatments, particularly in children, has the advantages of avoiding further scarring due to skin harvesting as well as the associated risks of anesthesia and bleeding. However, the drawbacks include a significantly prolonged treatment period, continued burden of wound care, and increased anxiousness experienced by the child’s parents until the wound heals.

The ASCS system that delivers epidermal cells to burn wounds allows treatment of a wide area with a small amount of skin graft [2]. Although full-thickness burns require combination with autologous skin grafts, DDB can be treated with ASCS alone. The advantage of ASCS is that it can significantly reduce the area of skin harvesting, making it particularly effective in the treatment of burns in children, where it is desirable to minimize the scarring. However, detailed reports on ASCS monotherapy for children are scarce. In a case series using ASCS in children, Wala et al. presented the results of three cases treated with ASCS monotherapy [9]. They concluded that ASCS monotherapy can be safely used in pediatric burn patients with relatively small burn areas and non-full-thickness burns, but its indication should be carefully considered [9]. Therefore, in the current study, we explored the early use of ASCS monotherapy for pediatric patients hospitalized with extensive burns and examined the treatment course and characteristics.

In all three cases in the current report, the mechanism of injury was scald burn, and there were no full-thickness burns. The burn area at the first visit ranged from 15% to 22.5%. Most of the burn area was SDB, while the total DDB area treated with ASCS was limited, ranging from 4% to 5% and distributed across multiple separate locations. When burn wounds are spread across multiple locations, changing the dressings is burdensome even if the wounds are small. Particularly in children who may be restless or too young to follow instructions, several people may need to be involved in wound care. Therefore, compared to conservative treatment alone, we believe that shortening the wound treatment period with ASCS has the advantage of reducing the burden on medical professionals and families involved in wound care and on the patients themselves. In our cases, there was a waiting period of up to 14 days before using ASCS in order to determine the burn depth and area. However, if the skin sampling area required to create the ASCS suspension was not significantly different, using it earlier may have reduced the number of sedation procedures before ASCS and shortened the treatment period.

In adults, the reported healing time for DDB wounds treated with ASCS monotherapy is approximately 13 days after surgery [10]; however, in pediatric patients, healing reportedly takes 39-50 days after injury [9]. Similarly, the cases in the current report had a healing period of 41-45 days after injury. We speculate that the extended healing period is due to the challenge of ensuring children strictly adhere to rest post surgery. Excessive stimulation may inhibit the ASCS engraftment, as it can easily be damaged during the early, fragile period. Future issues to be addressed include protocols for rest and wound dressing that would promote a shortened healing period.

Regarding the condition of postoperative scars, an extended healing period often results in inflammation and thickening of the wound. Additionally, wounds located in areas of movement initially appear flat post-epithelialization, but may thicken over time. In the two cases where steroid tape could be applied post epithelialization, the VSS outcomes were consistent with those reported in previous studies [9]. However, in the case where the application of the tape was not feasible, the VSS results were unsatisfactory. Given the higher incidence of hypertrophic scars in non-White populations [11], the use of steroid tape appears to be an effective intervention to enhance the improvement of scar quality more rapidly in these populations [5]. A challenge arises, however, in terms of patient compliance regarding younger children who may remove the steroid tape themselves. In addition, long-term follow-up is essential as the appearance of the scars may worsen due to the growth of the patient.

ASCS monotherapy for pediatric DDB offers several advantages, including expedited healing, reducing wound care burden, and minimizing scarring from skin harvesting. However, certain challenges remain with this therapy. The engrafted cells are fragile and may fall off due to mechanical trauma. Also, even though skin grafts are not required, the small amounts of skin needed for ASCS cannot be avoided. Other issues include postoperative scarring and the treatment cost. Currently, there are few reports on ASCS monotherapy in children, highlighting the need for continued accumulation of cases to refine the treatment protocols. Additionally, given the relative recency of ASCS’s introduction to Japan, this report offers only short-term observation; thus, long-term observation with extended follow-up to assess outcomes, including the impacts of growth, is necessary.

## Conclusions

The three cases described in this report of DDB in pediatric patients under the age of three years indicate that ASCS monotherapy offers significant benefits, such as expedited healing, decreased wound care burden, and minimized scarring from skin harvesting. Despite these advantages, some challenges remain, including the fragility of engrafted cells, the inevitable need for some degree of skin harvesting and postoperative scarring, and the treatment costs. This report of ASCS monotherapy in young patients contributes to the ongoing discussion on optimizing pediatric burn care, emphasizing the potential of ASCS monotherapy in mitigating the complexities of skin grafting. However, further research, case accumulation, and long-term follow-up are essential.
